# Altered dNTP pools accelerate tumor formation in mice

**DOI:** 10.1093/nar/gkae843

**Published:** 2024-10-03

**Authors:** Phong Tran, Pradeep Mishra, Leonard G Williams, Roman Moskalenko, Sushma Sharma, Anna Karin Nilsson, Danielle L Watt, Pernilla Andersson, Anders Bergh, Zachary F Pursell, Andrei Chabes

**Affiliations:** Department of Medical Biochemistry and Biophysics, Umeå University, Linnaeus väg 6, Umeå, SE 90736, Sweden; Department of Medical Biochemistry and Biophysics, Umeå University, Linnaeus väg 6, Umeå, SE 90736, Sweden; Department of Biochemistry and Molecular Biology, Tulane University School of Medicine, 1430 Tulane Ave, New Orleans, LA 70112, USA; Tulane Cancer Center, Tulane University School of Medicine, 1430 Tulane Ave, New Orleans, LA 70112, USA; Department of Pathology, Sumy State University, Kharkivska st. 116, Sumy 40007, Ukraine; Department of Medical Biochemistry and Biophysics, Umeå University, Linnaeus väg 6, Umeå, SE 90736, Sweden; Department of Medical Biochemistry and Biophysics, Umeå University, Linnaeus väg 6, Umeå, SE 90736, Sweden; Department of Medical Biochemistry and Biophysics, Umeå University, Linnaeus väg 6, Umeå, SE 90736, Sweden; School of Medicine and School of Dental Medicine, UConn Health, 300 UConn Health Blvd, Farmington, CT 06030, USA; Pathology Unit, Department of Medical Biosciences, Umeå University, Daniel Naezéns väg 6M, Umeå, SE 90737, Sweden; Pathology Unit, Department of Medical Biosciences, Umeå University, Daniel Naezéns väg 6M, Umeå, SE 90737, Sweden; Department of Biochemistry and Molecular Biology, Tulane University School of Medicine, 1430 Tulane Ave, New Orleans, LA 70112, USA; Tulane Cancer Center, Tulane University School of Medicine, 1430 Tulane Ave, New Orleans, LA 70112, USA; Department of Medical Biochemistry and Biophysics, Umeå University, Linnaeus väg 6, Umeå, SE 90736, Sweden

## Abstract

Alterations in deoxyribonucleoside triphosphate (dNTP) pools have been linked to increased mutation rates and genome instability in unicellular organisms and cell cultures. However, the role of dNTP pool changes in tumor development in mammals remains unclear. In this study, we present a mouse model with a point mutation at the allosteric specificity site of ribonucleotide reductase, RRM1-Y285A. This mutation reduced ribonucleotide reductase activity, impairing the synthesis of deoxyadenosine triphosphate (dATP) and deoxyguanosine triphosphate (dGTP). Heterozygous *Rrm1*^+/Y285A^ mice exhibited distinct alterations in dNTP pools across various organs, shorter lifespans and earlier tumor onset compared with wild-type controls. Mutational spectrum analysis of tumors revealed two distinct signatures, one resembling a signature extracted from a human cancer harboring a mutation of the same amino acid residue in ribonucleotide reductase, *RRM1*^Y285C^. Our findings suggest that mutations in enzymes involved in dNTP metabolism can serve as drivers of cancer development.

## Graphical Abstract

**Graphical Abstract:**
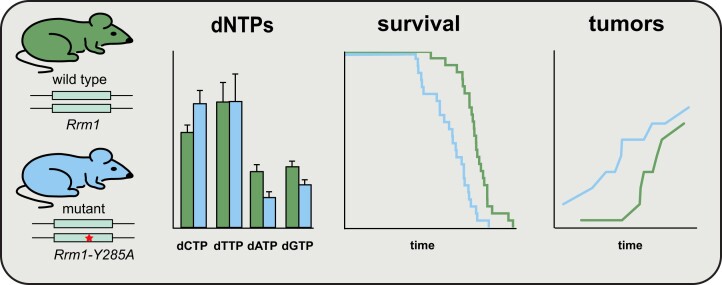

## Introduction

DNA replication fidelity relies on three critical determinants: nucleotide selectivity of replicative DNA polymerases, exonucleolytic proofreading of mismatches by polymerases and the DNA mismatch repair (MMR) system (1). Notably, defects in the latter two determinants are strongly associated with human cancers. In the 1990s, inactivation or mutations in MMR genes were found in colorectal cancers from Lynch syndrome patients, and similar mutations were subsequently identified in lung, gastric and endometrial cancers (2). Two decades later, studies showed that germline mutations in the proofreading domains of replicative polymerases Pol δ and Pol ϵ predispose to cancer and that somatic mutations in the Pol ϵ proofreading domain occur in multiple sporadic tumors (3).

However, the extent to which defects in the first determinant of DNA replication fidelity—nucleotide selectivity—are linked to cancer development remains less established. Decreased nucleotide selectivity can arise from mutations affecting the active site of DNA polymerases. For instance, a mouse strain harboring a heterozygous mutation in the active site of Pol δ (*Pold1*^+/L604K^) exhibited reduced genomic stability and accelerated tumorigenesis (4). Furthermore, specific residues (L606 and R689) impacting the nucleotide selectivity of human Pol δ have been implicated in several human cancers (5). Interestingly, mutations affecting the polymerase active sites of Pol δ and Pol ϵ are less common in cancers compared with those affecting proofreading or MMR. This discrepancy may be attributed to the fact that mutations decreasing nucleotide selectivity also lead to a decrease in polymerase activity. Notably, decreased nucleotide selectivity can arise not only from mutations in DNA polymerases but also from changes in the pool of deoxyribonucleoside triphosphates (dNTPs).

Mammalian cells produce and maintain dNTP pools using both *de novo* synthesis and salvage pathways (6). In the *de novo* pathway, ribonucleotide reductase (RNR) reduces the four canonical ribonucleoside diphosphates to their respective deoxyribonucleoside diphosphates (dNDPs). Deoxyadenosine diphosphate (dADP), deoxycytidine diphosphate (dCDP) and deoxyguanosine diphosphate (dGDP) are directly phosphorylated to form dNTPs, whereas the production of thymidine triphosphate (dTTP) requires several additional enzymes, including dUTPase, deoxycytidine monophosphate (dCMP) deaminase, thymidylate synthase and thymidylate kinase (7). Multiple enzymes within the purine and pyrimidine biosynthesis pathways regulate the concentrations of nucleoside diphosphates (NDPs)—the substrates for RNR. In the salvage pathways, deoxyribonucleosides (dNs) are phosphorylated by dedicated deoxyribonucleoside kinases (such as thymidine kinases 1 and 2, deoxyguanosine kinase and deoxycytidine kinase) to form deoxyribonucleoside monophosphates (dNMPs). Subsequently, these dNMPs are further phosphorylated to generate dNTPs. When dNTP levels exceed cellular requirements, the dNTP triphosphohydrolase SAM domain and HD domain-containing protein 1 degrades excess dNTPs to dNs (7). Mutations affecting any of these enzymes can alter dNTP pools and subsequently negatively impact replicative DNA polymerases.

The balance of dNTPs in rapidly proliferating mammalian cells is primarily governed by the allosteric regulation of RNR, which consists of a large catalytic subunit (RRM1) with two allosteric sites (specificity and activity) and a small subunit (RRM2 or RRM2B) with a tyrosyl radical required for catalysis (8). The allosteric *activity* site modulates the overall enzyme activity. Binding of ATP to this site stimulates the overall activity, while binding of dATP inhibits it. The allosteric *specificity* site, on the other hand, determines the relative production of different dNTPs. When ATP binds to the specificity site, it stimulates the production of dCDP and deoxyuridine diphosphate (dUDP), both of which are used to produce dTTP. Binding of dTTP to this site stimulates the production of dGDP, whereas binding of dGTP stimulates the production of dADP. The specificity site (residues 283–295) is highly conserved and identical in budding yeast, mouse and human RNRs, with the same amino acid residue numbers in these three species (Supplementary Figure S1A). Several residues within this site—A283, R284, Y285, D287, R293 and P294—have been identified as mutated in human cancer samples according to the cBioportal for Cancer Genomics. Previously, we demonstrated that mutations in the allosteric specificity site of budding yeast RNR lead to different dNTP pool imbalances causing increased mutation rates (9). Subsequently, an unbiased screen identified 24 mutant alleles in budding yeast RNR that conferred mutator phenotypes (10). Four of the yeast RNR residues identified in this screen (A245, R256, A283 and Y285) have also been found mutated in human *RRM1* in cancer samples (cBioportal for Cancer Genomics and/or the Catalogue of Somatic Mutations in Cancer databases).

The role of mutations causing altered dNTP pools as potential initiators of cancer remains uncertain. To investigate this, we introduced the Y285A mutation in the region encoding the allosteric specificity site of the mouse *Rrm1* gene. We selected this substitution because a haploid budding yeast strain carrying the *rnr1*-Y285A mutation was viable and exhibited normal proliferation, despite a pronounced dNTP pool imbalance (∼20-fold increased deoxycytidine triphosphate (dCTP) and 17-fold increased dTTP levels, alongside normal dATP and dGTP levels), and a substantial ∼13-fold increase in mutation rate (9). In this work, surprisingly, the same mutation in mice (*Rrm1*^Y285A/Y285A^) resulted in embryonic lethality. However, the heterozygous mice (*Rrm1*^+/Y285A^) were viable and exhibited tissue-specific dNTP pool imbalances, reduced lifespan and earlier onset of tumors. Our analysis of tumor mutational spectra revealed two different signatures, one closely resembling a signature from a human cancer with a mutation at the same residue in RRM1 (Y285C). Measurements of RRM1-Y285A and RRM1-Y285C proteins activity demonstrated decreased efficiency in dADP and dGDP synthesis. These findings suggest that perturbations in dNTP pools may act as drivers of cancer development.

## Materials and methods

### Reagents

NativeMark™ Unstained Protein Standard (Thermo Fisher Scientific, Cat#LC0725); 10% neutral-buffered formalin (Sigma–Aldrich, Cat#HT501128); Mayers HTX (Histolab, Cat#01820); Eosin Y (Histolab, Cat#01650); high pressure liquid chromatography (HPLC) column 4.6 × 150 mm Sunshell C18-WP 2.6 (ChromaNik Technologies Inc., Osaka, Japan, Cat#CW6471); and TwoMP mass photometer (Refeyn Ltd, https://www.refeyn.com/twomp-mass-photometer).

### Biological resources

Escherichia coli BL21 (DE3) (Sigma–Aldrich, Cat#69450-M); bacterial plasmid pD441-H6SUMO (doi.org/10.1074/jbc.M117.776310); mouse: *Rrm1^+/Y285A^*: C; B6-*Rrm1^tm1Ach^*.

### Statistical analysis

Data were analyzed with the Mann–Whitney *U*-test or unpaired Student’s *t*-test with Welch’s correction and graphed with GraphPad Prism 8.0.2 software. Kaplan–Meier curves were compared using the (log-rank) Mantel–Cox test. All tests had a significance level of *P* < 0.05.

### Web sites/database referencing

- Whole exome sequencing raw datasets (SRA accession number PRJNA1114130);
- Burrows-Wheeler Aligner (doi.org/10.48550/arXiv.1303.3997; http://bio-bwa.sourceforge.net);
- GATK4 (ISBN-13: 978–1491975190; https://gatk.broadinstitute.org/hc/en-us);
- VarScan2 (https://doi.org/10.1002/0471250953.bi1504s44; http://varscan.sourceforge.net);
- SAMtools (doi.org/10.48550/arXiv.1303.3997; http://samtools.sourceforge.net);
- BCFtools (doi.org/10.1093/gigascience/giab008; https://www.htslib.org);
- R package: Tidyverse (doi.org/10.21105/joss.01686; https://tidyverse.tidyverse.org/index.html);
- R package: MutationalPatterns (https://doi.org/10.1186/s13073-018-0539-0; https://bioconductor.org/packages/release/bioc/html/MutationalPatterns.html);
- R package: pheatmap (https://cran.r-project.org/package=pheatmap);
- R package: NMF (doi.org/10.1186/1471–2105-11–367; http://cran.r-project.org/package=NMF); and
- DiscoverMP software, Refeyn Ltd (https://www.refeyn.com/software-release-updates).

### Expression and purification of recombinant human RNR

Plasmid pD441-H6SUMO (11) was used to construct the pD441SM6H-hRRM1 and pD441SM6H-hRRM2 plasmids that were used to produce the human RRM1 (hRRM1) and human RRM2 (hRRM2) proteins as previously described (11,12). The Y285A and Y285C mutations were introduced into the pD441SM6H-hRRM1 plasmid by site directed mutagenesis using oligonucleotides hRRM1-Y285A-F, hRRM1-Y285C-F and hRRM1-285-R (Supplementary Table S1) and verified by DNA sequencing. The expression and purification processes for hRRM1-Y285A and hRRM1-Y285C were conducted similarly to those for the wild-type (WT) hRRM1 protein. Protein purity was analyzed by sodium dodecyl sulfate–polyacrylamide gel electrophoresis (Supplementary Figure S1D).

### RNR activity measurements

The enzymatic activity of recombinant hRRM1, hRRM1-Y285A and hRRM1-Y285C was measured using either CDP only or all four NDPs (CDP, UDP, GDP and ADP) as substrates. The CDP reductase assay was performed as described previously (13). Briefly, 50-μl reaction mixtures were prepared containing 40 mM Tris–HCl (pH 7.6), 200 mM KCl, 5 mM dithiothreitol (DTT), 20 μM FeCl_3_, 5 mM MgCl_2_, 0.5 mM ^3^H-CDP and 2 mM ATP, along with allosteric effectors (dTTP, dGTP and dATP) at specified concentrations. The reactions were initiated by adding 1 μM hRRM1, hRRM1-Y285A or hRRM1-Y285C, along with an excess of hRRM2 (5 μM), and carried out at 37°C for 15 min.

In the assay utilizing all four NDP substrates, the reaction mixture contained 0.2 mM of each NDP, along with four allosteric effectors (ATP 2 mM, dATP 14 μM, dGTP 6 μM and dTTP 30 μM). The reaction was started by adding 1 μM hRRM1, hRRM1-Y285A, hRRM1-Y285A, a 1:1 mixture of hRRM1/hRRM1-Y285A or a1:1 mixture of hRRM1/hRRM1-Y285C, along with an excess of hRRM2 (5 μM). The assay was performed at 37°C for 5–30 min, followed by termination at 90°C for 2 min. The generated dNDPs were analyzed by HPLC using a 4.6 × 150 mm Sunshell C18-WP 2.6 μm column (ChromaNik Technologies Inc., Osaka, Japan) at a flow rate of 1 ml/min. The mobile phase comprised 50% buffer A (23 gm/l KH_2_PO_4_ + 5% acetonitrile + 0.7 g/l tetrabutylammonium bromide; pH5.6) and 50% buffer B (5% acetonitrile + 0.7 gm/l tetrabutylammonium bromide). Identification of dNDPs was done by comparing with standards, and quantification was performed at 260 nm using peak area.

### Mass photometry

Mass photometry data were acquired on a Refeyn Two MP mass photometer. The hRRM1, hRRM1-Y285A or hRRM1-Y285C proteins at a concentration of 1 μM were incubated in mass photometry buffer (50 mM Tris–HCl, 100 mM KCl and 5mM MgCl_2_) with or without allosteric effectors (100 μM dTTP, 100 μM dGTP and 2 mM ATP) for 5 min at room temperature. Proteins were diluted immediately prior to measurements in a buffer containing allosteric effectors to 10 nM (for dTTP and dGTP analysis) and 20 nM (for ATP analysis). Movies were acquired for 60 s and analyzed using DiscoverMP software. The contrast value was converted into molecular mass using a calibration curve obtained with NativeMark™ Unstained Protein Standard (catalog number: LC0725, Thermo Fisher Scientific, USA).

### Generation of transgenic mice

*Rrm1^+/Y285A^* mice were constructed at inGenious Targeting Laboratory. The genomic sequence from exon 8 to exon 15 of the *Rrm1* gene was subcloned into a target vector using homologous recombination techniques. The vector (KnockIn vector) also carried a Neomycin (Neo) cassette (FRT-PGK-gb2-Neo-LoxP-FRT) inserted in the intron sequence between exons 8 and 9. In exon 9, the TA nucleotides in the codon that encode for amino acid 285 were mutated to GC, thus changing tyrosine 285 to alanine. The KnockIn vector was linearized with *Not*I and transfected into C57BL/6 embryonic stem cells by electroporation. Neo-resistant clones were injected into the blastocysts of Balb/c surrogate mice to obtain chimeric transgenic mice, and the resulting chimeras with a high percentage of coat color were mated to C57BL/6 FLP mice to remove the Neo cassette, leaving an flippase recognition target (FRT) site (Supplementary Figure S1A).

### Maintenance of transgenic mice

Animal experiments were approved by the Animal Review Board at the Court of Appeal of Northern Norrland in Umeå (#A29-2018) and complied with the rules and regulations of the Swedish Animal Welfare Agency and with the European Communities Council Directive of 22 September 2010 (2010/63/EU). All efforts were made to minimize the animals’ suffering and to reduce the number of animals used. All mice were maintained at Umeå Centre for Comparative Biology under pathogen-free conditions. Mice were housed in a 12-h dark/light cycle environment with *ad libitum* access to food and water. The genotype of all animals was determined by polymerase chain reaction analysis of genomic DNA (gDNA) extracted from ear punch biopsies and utilized the presence of the remaining FRT site. Two primers were used: NDEL1 and GT1 (Supplementary Table S1). The WT band was 345 bp and the mutant band was 424 bp.

### dNTP pool measurements in mouse embryos and organs

To obtain embryos, females were paired individually overnight with males and inspected for vaginal plugs the next morning, at which point the embryos were considered to be at E0.5. At E13.5, the pregnant females were euthanized by cervical dislocation and the whole embryos were immediately isolated. The tails were taken for genotyping, and the embryos were put in Eppendorf tubes containing 700 μl ice-cold 12% (w/v) trichloroacetic acid with 15 mM MgCl_2_ and snap-frozen in liquid nitrogen and stored at −80°C. For isolation of organs, mice were euthanized by cervical dislocation, organs were immediately isolated and put in Eppendorf tubes containing 500 μl ice-cold 30% (w/v) trichloroacetic acid with 45 mM MgCl_2_ and snap-frozen in liquid nitrogen and stored at −80°C. Nucleotide extraction was performed as described (14). Samples were cleaned with solid-phase extraction columns and analyzed by HPLC as described (15,16).

### Histopathologic analysis

Mice were euthanized by carbon dioxide inhalation followed by necropsy. Dissected organs and bodies were fixed in 10% neutral-buffered formalin (HT501128, Sigma–Aldrich) for 24 h and stored in 70% ethanol. Organs were sampled, trimmed and embedded in paraffin blocks. Serial sections (∼4 μm/section) from the paraffin blocks were stained with hematoxylin (Mayers HTX, Histolab) and 0.2% eosin Y (Histolab). The stained sections were scanned on a 3D Histech Pannoramic 250 Flash III scanner, and the digital sections were examined by two pathologists in a blinded manner.

### Whole exome sequencing

gDNA from WT and *Rrm1*^Y285A^ tumors and normal tissues was extracted using 10 mM Tris-HCl, pH 7.5; 125 mM NaCl; 10 mM EDTA; 1% SDS (TNES) lysis buffer followed by phenol:chloroform extraction and ethanol precipitation, and finally resuspended in 10 mM Tris-HCl; 1mM EDTA (TE) buffer. gDNA was prepared and sent to BGI Tech Solutions (Hong Kong) for whole exome sequencing (WES). Sequencing libraries were prepared using Agilent SureSelect Mouse All Exon kit and sequenced on the Illumina Hiseq4000 platform.

### WES data processing and variant calling

Raw FastQ reads were mapped to the mouse genome (UCSC GRCm38/mm10, December 2011) with BWA-MEM v0.7.15 and duplicate reads were flagged with MarkDuplicates from GATK v4.1.8.1 (17,18). The mean depth of coverage of cleaned, deduplicated, mapped reads over the target region for all samples was 96.5× (range 82.2–120.8×) as calculated with SAMtools v1.12 (19). Somatic short variants were called with Varscan2 in tumor-normal mode (Basic Protocol 2) (20,21) using a strain-similar WT normal from the cohort. *Post hoc* variant filtering was performed to remove common variants among the cohort that were likely to be false positive genetic background variants. Briefly, BCFtools was used to determine variants occurring in at least two samples which were then compiled into a sites-only Variant Call Format (VCF). These variants were then retroactively masked from the original sample VCFs before downstream analysis (GenomicDataCommons: NIH/NCI Genomic Data Commons Access; R package v.1.14.0, 2020).

### Mutation analysis

Sample mutation levels were calculated with BCFtools and the Tidyverse suite of R packages (22). Student’s *t*-test was used to test for a difference in mean mutation levels between WT and *Rrm1*^Y285A^ mouse tumors. Data for the human tumor with the analogous *RRM1*^Y285C^ mutation were retrieved from the Genomic Data Commons Data Portal (https://portal.gdc.cancer.gov) and additional metadata were retrieved with the GenomicDataCommons R package (23).

Mutational signature analysis was performed using the R packages MutationalPatterns, NMF and pheatmap (24,25). Non-negative matrix factorization (NMF) was used to determine the optimal number of mutational signatures (the NMF rank) to extract. A stable rank of 2 was chosen based on the cophenetic correlation coefficient and residual sum of squares estimation parameters. Original sample mutation spectra were then reconstructed using the *de novo* NMF signatures to obtain the cosine similarities for assessing signature robustness. Samples were hierarchically clustered based on their reconstructed contributions from the two *de novo* signatures.

## Results

### Homozygous *rrm1*-Y285A mutation causes embryonic lethality

The mouse *Rrm1*^+/Y285A^ strain was generated by targeted replacement of exons 8–15 of the *Rrm1* gene with a construct containing a TA to GC mutation in exon 9, resulting in the conversion of tyrosine 285 to alanine (Supplementary Figure S1B). Attempts to obtain homozygous *Rrm1*^Y285A/Y285A^ animals by crossing heterozygous *Rrm1*^+/Y285A^ mice were unsuccessful (Supplementary Figure S1C), and no embryos homozygous for *rrm1-*Y285A mutation were detected as early as embryonic day E6.5.

**Figure 1.**
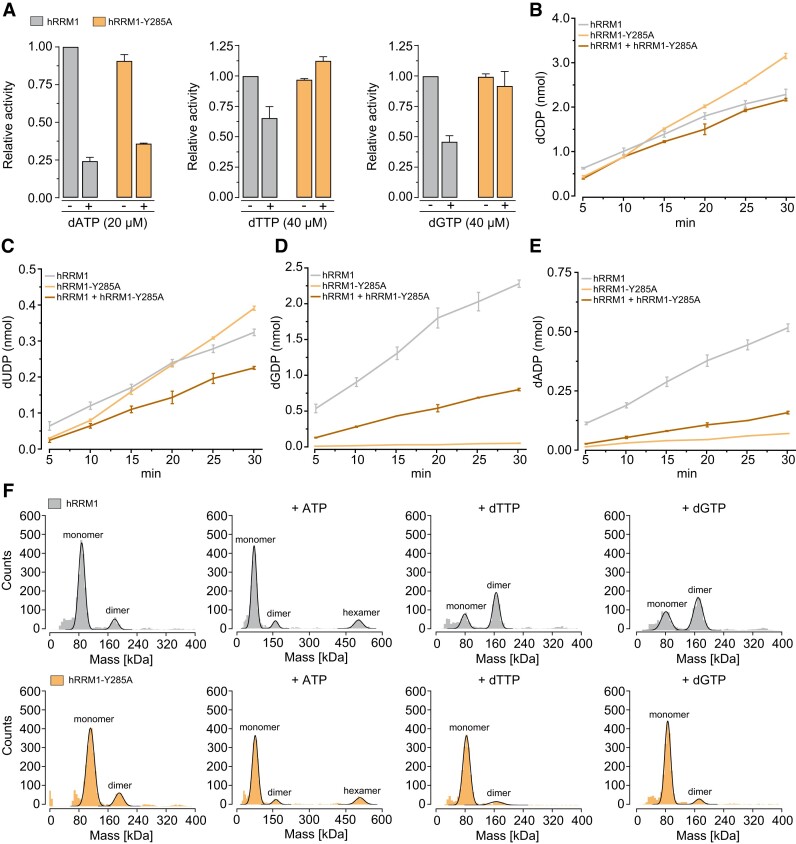
Activity and subunit composition of hRRM1 and hRRM1-Y285A in the presence of different allosteric effectors. (**A**) Relative CDP-reducing activity of hRRM1 and hRRM1-Y285A proteins in the presence of hRRM2 with 2 mM ATP, with or without 20 μM dATP, 40 μM dTTP or 40 μM dGTP. Activity of hRRM1 in the presence of ATP is normalized to 1. (**B–E**) Activity of hRRM1, hRRM1-Y285A and a 1:1 mixture of hRRM1/hRRM1-Y285A protein mixture in the presence of hRRM2 with four substrates (200 μM each of CDP, UDP, GDP and ADP) and four allosteric effectors at physiological concentrations (2 mM ATP, 30 μM dTTP, 14 μM dATP and 6 μM dGTP) over a 30-min time course. (**F**) Mass photometry analysis of the subunit composition of hRRM1 and hRRM1-Y285A proteins in the presence or absence of positive allosteric effectors (2 mM ATP, 100 μM dTTP and 100 μM dGTP).

To understand the cause of this lethality, we assessed the activity of the recombinant hRRM1 protein, which shares 98% sequence identity with the mouse counterpart and has an identical total number of amino acid residues. In all assays, we used an excess of hRRM2. Both WT and hRRM1-Y285A proteins were active in reducing CDP to dCDP in the presence of ATP, and both were inhibited by dATP (Figure 1A), consistent with allosteric regulation of RNR.

The CDP-reducing activity of the WT protein was inhibited by dTTP and dGTP, as expected due to their binding to the allosteric specificity site of hRRM1, shifting its activity from CDP reduction to GDP and ADP reduction, respectively (Figure 1A). In contrast, the CDP-reducing activity of hRRM1-Y285A remained unaffected by dTTP or dGTP, indicating no shift from CDP reduction to GDP or ADP reduction.

We then examined the activity of hRRM1, hRRM1-Y285A and a 1:1 mixture of hRRM1/hRRM1-Y285A in RNR assays containing all four substrates (CDP, UDP, GDP and ADP) and all four allosteric effectors (ATP, dATP, dTTP and dGTP) at their physiological concentrations (16). While the production of dCDP and dUDP was similar across all three cases (Figure 1B and C), the production of dGDP and dADP was severely impaired in the hRRM1-Y285A assay and reduced in the hRRM1/hRRM1-Y285A mixture (Figure 1D and E).

RRM1 becomes active after forming dimers driven by binding of dTTP, dGTP or (d)ATP at the allosteric specificity site (8). At higher ATP concentrations, its binding to the allosteric activity site drives the formation of hexamers, increasing the overall RNR activity. Mass photometry analysis demonstrated that both hRRM1 and hRRM1-Y285A formed hexamers in the presence of ATP, consistent with RNR activity measurements (Figure 1F). However, dTTP- and dGTP-dependent dimerization occurred only in hRRM1 protein, not in RRM1-Y285A (Figure 1F), again in agreement with the RNR activity measurements. The allosteric regulation of RRM1-Y285C closely resembled that of RRM1-Y285A, but its overall activity was lower (Supplementary Figure S2). Taken together, these data suggest that embryonic lethality of *Rrm1*^Y285A/Y285A^ mice likely results from impaired dGDP and dADP production.

### Heterozygous *Rrm1*^+/Y285A^ mice exhibit varied dNTP pool imbalances across different organs

**Figure 2.**
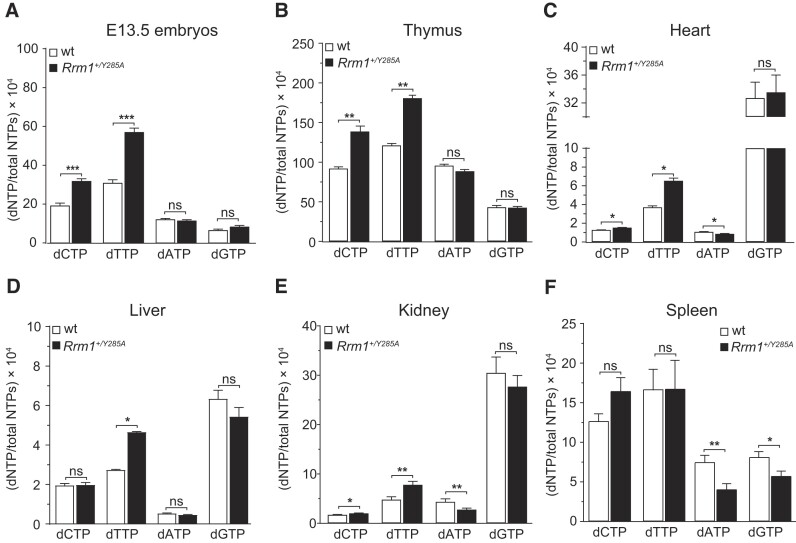
dNTP pools in various organs of heterozygous *Rrm1*^+/Y285A^ mice. (**A**) E13.5 embryos, *n* = 6 for WT, *n* = 12 for *Rrm1*^+/Y285A^. (**B**) Thymus, *n* = 6. (**C**) Heart, *n* = 4. (**D**) Liver, *n* = 4. (**E**) Kidney, *n* = 9 for WT, *n* = 13 for *Rrm1*^+/Y285A^. (**F**) Spleen, *n* = 10. dNTPs were normalized to total NTPs. Data are presented as mean ± standard error of the mean. Statistical significance was determined using Mann–Whitney *U*-tests, with *** indicating *P* < 0.001, ** indicating *P* < 0.01, * indicating *P* < 0.05 and ‘ns’ indicating non-significant.

Heterozygous *Rrm1*^+/Y285A^ mice were born healthy without discernible phenotypes. Analysis of dNTP pools in E13.5 embryos revealed an approximately 2-fold increase in dCTP and dTTP pools compared with WT mice, with no significant changes observed in dATP and dGTP pools (Figure 2A). This trend resembled the imbalance previously observed in *rnr1*-Y285A budding yeast (9), albeit to a lesser degree.

Examination of dNTP pools in various organs of 13-week-old animals showed a mixed picture. In the thymus, dNTP pool imbalances in *Rrm1*^+/Y285A^ mice were similar to those found in embryos (Figure 2B). In the heart, liver and kidney, one or both pyrimidine dNTPs were increased compared with WT mice (Figure 2C–E). Interestingly, in the heart, kidney and spleen, one or both purine dNTPs were decreased compared with WT mice (Figure 2C, E and F). We conclude that *rrm1*-Y285A mutation has a dominant effect, and the observed dNTP pool imbalance aligns with the activity measurements of the hRRM1/hRRM1-Y285A protein mixture.

The variations observed in dNTP pool levels could be attributed to differences in cell proliferation rates across organs. Highly proliferative tissues like embryos and the thymus, with abundant RNR protein expression, might maintain dATP and dGTP concentrations in *Rrm1*^+/Y285A^ mice comparable to WT animals. However, due to relatively higher production of pyrimidine dNTPs compared with purine dNTPs by RRM1/RRM1-Y285A proteins (Figure 1B–E), dCTP and dTTP concentrations were elevated in embryos and thymuses of *Rrm1*^+/Y285A^ mice.

Organs like the heart, liver, kidney and, to a lesser extent, spleen contain larger proportion of non-dividing, terminally differentiated cells, where dNTP pools are low, and their balance is different from that of actively proliferating cells. The unusually high proportion of dGTP in the hearts, livers and brains of WT mice has been previously documented (26,27) and is most likely related to the specific binding of dGTP to the mitochondrial protein NDUFA10 (27). Other organs with many non-dividing cells and abundant mitochondria likely also have a high proportion of dGTP for the same reason. The decreased levels of one or both purine dNTPs in the heart, kidney and spleen of *Rrm1*^+/Y285A^ mice could be due to insufficient amounts of RRM1/RRM1-Y285A proteins as well as insufficient salvage processes to maintain adequate dATP and dGTP levels. Thus, in *Rrm1*^+/Y285A^ mice, dNTP imbalances can manifest both as elevated levels of dCTP and dTTP and as reduced levels of dGTP and dATP.

### Mice with altered dNTP pools demonstrate shortened lifespans and earlier onset of tumors

**Figure 3.**
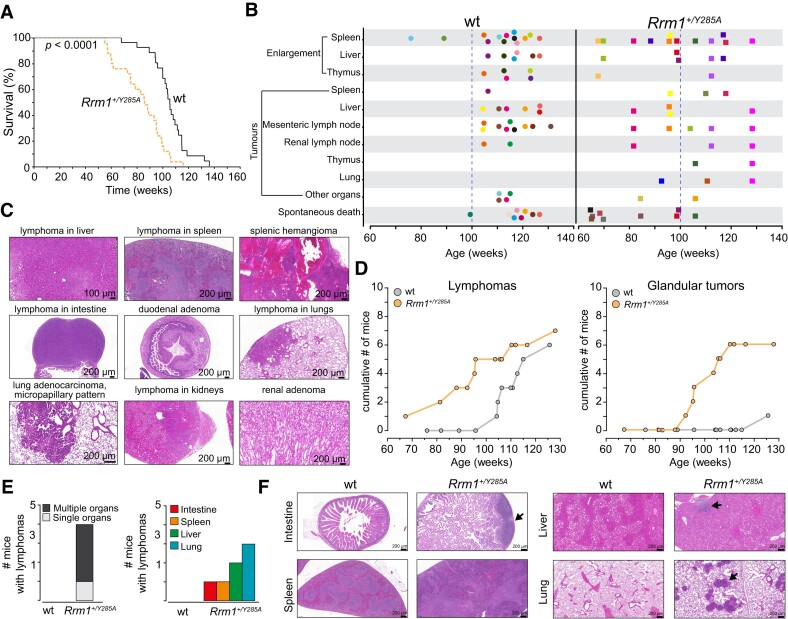
Altered dNTP pools lead to a shortened lifespan and earlier onset of cancers. (**A**) Kaplan–Meier survival curve spanning 150 weeks, compiled from 27 WT and 26 *Rrm1*^+/Y285A^ mice. Statistical analysis was performed using the Mantel–Cox test. (**B**) Diagram depicting observed abnormalities in mice during the lifespan experiment plotted against the ages at which the abnormalities were detected. Each color represents an individual mouse. (**C**) Representative hematoxylin and eosin (HE) stained sections of indicated tumors. (**D**) Cumulative numbers of mice with lymphomas and glandular tumors (adenomas and adenocarcinomas) compiled from 12 *Rrm1*^+/Y285A^ and 13 age-matched WT mice euthanized during the lifespan study. (**E**) The number of 1-year old mice with lymphoma at one or multiple locations, compiled from 8 WT and 9 *Rrm1*^+/Y285A^ mice. (**F**) Representative HE-stained sections of the intestine, lung, liver and spleen of mice in (E). Arrows indicate lymphocytic infiltrates. Scale bar: 200 μm. Note the distinct borders between the white and red pulps in the WT spleen section, which are diminished in the *Rrm1*^+/Y285A^ spleen section.

The *Rrm1*^+/Y285A^ (*n* = 26) mice showed a significantly reduced lifespan compared with WT littermates (*n* = 27), with a median survival of 96 weeks compared with 117 weeks for WT mice (Figure 3A). Mice involved in the lifespan study either died spontaneously or were euthanized upon reaching humane endpoints. Notably, both WT and *Rrm1*^+/Y285A^ mice lived without any noticeable abnormalities for up to 1 year. However, after the first year, 20% of the *Rrm1*^+/Y285A^ mice died within a 2-month period (between weeks 60 and 68), whereas 20% of the WT control mice died over a period of 9 months (between weeks 75 and 105).

Nearly all mice included in the lifespan analysis exhibited various abnormalities, such as organ enlargement and tumors, at some stage in their lifetimes. However, *Rrm1*^+/Y285A^ mice began to display these phenotypes earlier than WT mice, who were largely free of such phenotypes up 100 weeks (Figure 3B). Specifically, eight of the *Rrm1*^+/Y285A^ mice died spontaneously before reaching 100 weeks, whereas only one of the WT mice did so over the same time period (Figure 3B). Before reaching 100 weeks, seven of the *Rrm1*^+/Y285A^ mice showed splenomegaly, whereas only two of the WT exhibited this condition. Liver enlargement was observed in three of the *Rrm1*^+/Y285A^ mice, and thymus enlargement was seen in one. In contrast, none of the WT mice displayed liver or thymus enlargement. Additionally, tumors of the spleen, liver, lung and lymph nodes were only observed in *Rrm1*^+/Y285A^ mice within the first 100 weeks (Figure 3B). Conversely, tumor development in WT mice was typically observed after 100 weeks, consistent with the genetic background of this strain (28) (Figure 3B). Notably, thymus tumors were found exclusively in *Rrm1*^+/Y285A^ mice. Overall, these findings suggest that altered dNTP pools lead to the earlier onset of pathological phenotypes, some of which are specific to *Rrm1*^+/Y285A^ mice.

To understand the nature of histopathological disorders in the mice in the lifespan experiment, we performed hematoxylin and eosin staining of brain, heart, lung, kidney, liver, spleen and intestine samples (Figure 3C). There were no cancers detected in the brain or heart. Both WT and *Rrm1*^+/Y285A^ mice developed lymphomas in the spleen, liver, intestines and lungs. However, lymphomas developed in the *Rrm1*^+/Y285A^ mice as early as at 68 weeks, but only began to appear at 105 weeks in the WT mice (Figure 3D). Two lymphomas in kidneys were found only in the *Rrm1*^+/Y285A^ mice, suggesting that this is a unique cancer phenotype resulting from the *Rrm1-Y285A* mutation (Figure 3C).

Due to the low number of adenocarcinomas, we grouped them with adenomas into glandular tumors (Figure 3D). Apart from one case of pulmonary adenocarcinoma in the oldest WT mice, all of the adenomas were found only in the *Rrm1*^+/Y285A^ mice with an onset at ∼95 weeks. Two renal adenomas were observed only the *Rrm1*^+/Y285A^ mice, indicating tissue-specific susceptibility to altered dNTP pools.

Given that the *Rrm1*^+/Y285A^ mice in the lifespan experiment exhibited increased mortality after 60 weeks, we designed another experiment in which 8 WT and 9 *Rrm1*^+/Y285A^ mice were euthanized and examined at the same age of 52 weeks. While no abnormalities were observed in WT mice at this stage, four of the *Rrm1*^+/Y285A^ mice had lymphomas in one or multiple organs (Figure 3F). Among the *Rrm1*^+/Y285A^ mice that had lymphomas, three developed lymphomas in the lungs, two in the liver, one in the spleen and one in the intestine. These results confirm our observation that altered dNTP pools lead to earlier onset and higher incidence of lymphomas.

### Tumors from *Rrm1*^+/Y285A^ mice exhibit mutation signatures resembling human cancer with *RRM1-*Y285C mutation

Assessing mutation burden and signatures can provide valuable insights into the underlying causes and mechanisms of tumor development in both humans (29,30) and mouse models (31–34). WES was conducted on three tumors from WT mice, six tumors from *Rrm1*^+/Y285A^ mice, and three normal tissues (one from a WT mouse and two from *Rrm1*^+/Y285A^ mice). We focused on variants unique to individual samples for analysis.

**Figure 4.**
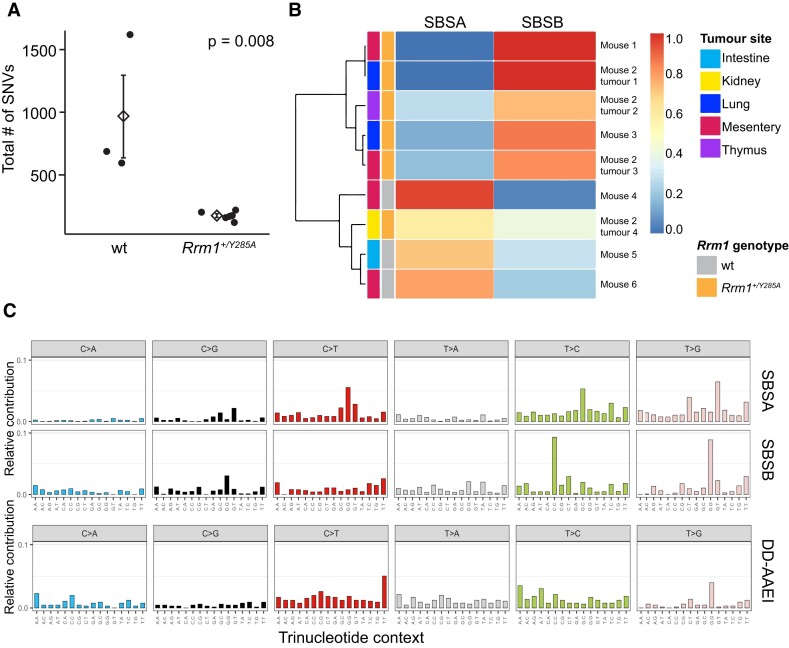
Mutation analysis of mouse and human tumors with mutations in RRM1 at position 285. (**A**) Mutation burden comparison between WT and *Rrm1*^+/Y285A^ mice. (**B**) Unsupervised hierarchical clustering of mutation signatures from WT and *Rrm1*^+/Y285A^ mouse tumors. SBSA and SBSB: single base substitutions A and B. (**C**) Mutational signatures observed in WT and Rrm1+/Y285A mouse tumors, alongside a mutation spectrum from a human patient (TCGA-DD-AAEI, The Cancer Genome Atlas) with an *RRM1*-Y285C mutation.

Contrary to expectations considering the accelerated mortality observed in *Rrm1*^+/Y285A^ mice, tumors from these mice did not show an overall increase in mutation burden compared with tumors from WT mice (Figure 4A). Surprisingly, there were actually 3–5-fold fewer variants in tumors from *Rrm1*^+/Y285A^ mice.

Two distinct mutation signatures, single base substitutions A and B (SBSA and SBSB), were extracted *de novo* from all tumor samples analyzed (Figure 4B and C). Unsupervised hierarchical clustering separated the samples into two distinct groups based on these signatures. Interestingly, this clustering also distinguished *Rrm1*^+/Y285A^ from WT samples (Figure 4B), with only *Rrm1*^+/Y285A^ samples showing high levels of SBSB and only WT samples showing high levels of SBSA. One *Rrm1*^+/Y285A^ sample (9R) displayed characteristics of both signatures. SBSB, enriched in all *Rrm1*^+/Y285A^ samples, is characterized by two distinct trinucleotide base pair substitutions: T > C transitions in CTC context and T > G transversions in GTG context. Notably, there is a single sample in The Cancer Genome Atlas (as of 1 April 2024) that contains a somatic mutation at the conserved Y285 residue, *RRM1*-Y285C. Analysis of the mutation spectrum from this patient (TCGA-DD-AAEI, Figure 4C) reveals two distinct peaks, one of which corresponds to T > G transversions in a GTG context.

## Discussion

This study reveals a novel aspect of tumorigenesis by demonstrating that altered dNTP pools in mice accelerate tumor development. Conventionally, increased mutation rates due to misincorporation of dNTPs during DNA replication are implicated in tumor development, particularly in cells with defective proofreading by replicative DNA polymerases or with defects in MMR (2,3). However, our findings suggest that in the *Rrm1*^+/Y285A^ mouse model, tumor development is not primarily driven by increased misincorporation of dNTPs. The dNTP pool imbalance caused by the *rrm1*-Y285A mutation is relatively mild, resulting in changes in purine-to-pyrimidine dNTP ratios of up to 2-fold at most, and it does not lead to an increased mutation burden in tumors. Intriguingly, the mutation burden in tumors of *Rrm1*^+/Y285A^ mice is actually lower than in tumors from WT mice.

Instead, we propose that increased tumorigenesis in *Rrm1*^+/Y285A^ mice is driven by replication stress, a phenomenon known to increase genomic instability (35,36). Our observations of impaired dGDP and dADP production by RRM1-Y285A-containing RNR (Figure 1A–E) and corresponding decreased purine dNTP levels in several organs of *Rrm1*^+/Y285A^ mice (Figure 2), suggest a potential mechanism underlying replication stress. In cell culture, reduced dNTP availability can impede replication fork progression, prompting replication fork pausing as cells attempt to adjust to the limited dNTP (37). If not resolved, the replication fork pausing can create long-term replication stress, ultimately resulting in genomic instability, capable of promoting tumor initiation and progression without necessarily increasing mutation rates. At the same time, a shift in dNTP pool balance can create a distinct mutation signature that differentiates cells with altered dNTP pools from normal cells, providing a potential biomarker for identifying tumors driven by altered dNTP pools. Interestingly, a reduction in mutation rate alongside an altered mutation signature has been observed in other contexts, such as in human cell lines and *Caenorhabditis elegans* with inactivated translesion polymerases Rev1 and Pol ζ (38,39). Additionally, BRCA1/2 mutations in human breast cancer tumors correlate with a characteristic structural variant signature and mutation signatures 3 and 8, without increasing the overall mutation burden (40).

The *rnr1*-Y285A mutation in budding yeast results in normal dATP and dGTP levels but leads to a dramatic increase in dCTP and dTTP levels, with elevations of ∼20-fold and 17-fold, respectively (9). Several factors may explain the differences in dNTP pools observed between mammals and yeast, despite the mutation affecting the same conserved RNR residue. In budding yeast, even minor DNA replication stress caused by DNA damage or a deficiency in one or several dNTPs triggers a checkpoint response. This response activates yeast RNR through multiple pathways, leading to a balanced expansion of the dNTP pools (41–43). We propose that the *rnr1*-Y285A mutation in budding yeast impairs the production of dATP and dGTP, causing replication stress and subsequent RNR activation and dNTP pool expansion. However, in the *rnr1*-Y285A strain, this expansion becomes imbalanced due to the reduced synthesis of purine dNTPs. Consequently, the high mutation rates observed in yeast with the *rnr1*-Y285A mutation are likely driven by dNTP pools that, while sufficient for replication, are highly imbalanced. These increased and imbalanced dNTP pools promote base substitutions as well as short insertions and deletions (44).

In contrast, mammalian cells do not activate RNR and do no undergo significant expansion of dNTP pools in response to DNA damage and replication stress (12). As a result, in mice, the same mutation in RNR leads to dNTP pools that are limiting for DNA replication and only mildly imbalanced. Reduced dNTP pools increase the efficiency of proofreading and reduce mutation rates. A similar situation might occur in budding yeast with an inactivated Dun1, a checkpoint kinase that is required for RNR activation. In *dun1* mutants, the dNTP pools are reduced (45), leading to a decrease in overall spontaneous mutagenesis (46). However, these yeast mutants exhibit increased levels of heteroallelic recombination (47). These observations suggest that reduced dNTP pools can increase genomic instability without necessarily elevating spontaneous mutagenesis.

A parallel can be also drawn to yeast strains with reduced levels of replicative polymerases. A 10-fold reduction in Pol α led to increased aneuploidy, chromosome loss and a 100-fold increase in a chromosome rearrangement reporter (48,49). This occurred alongside less than a 2-fold increase in mutagenesis at the CAN1 locus, which primarily measures base-pair substitutions and indels. This suggests that limiting replication factors, such as Pol α or dNTP pools, may increase genomic instability without substantially increasing base-pair substitutions and indels.

The fact that *Rrm1*-Y285A acts as a dominant mutation contrasts with mutations that inactivate the exonuclease activity of Pol δ *(Pold1*^e^) and Pol ϵ (*Pole*^e^), which are recessive mutants, and only *Pold1*^e/e^ or *Pole*^e/e^ homozygous mice display mutator phenotypes and cancers (50,51). Similarly, in the case of MMR deficiency, strong tumor predisposition is only observed in homozygous Msh2^–/–^, Msh6^–/–^, Mlh1^–/–^ and Pms2^–/–^ mutant mice (52–55). Unlike the *Rrm1^+/Y285A^* mice, heterozygous mice carrying mutation in MMR genes are indistinguishable from their WT controls with respect to survival and cancer phenotypes in most cases.

Another intriguing observation is that a unique mutation spectrum can be extracted from the *Rrm1^+/Y285A^* samples. This spectrum does not closely match any existing human tumor signatures and is largely defined by two major peaks, T > C-CTC and T > G-GTG (Figure 4C). Both of these errors can arise from misinsertions that are stabilized by the next nucleotide on either strand. For example, the T > G-GTG mutation could arise on the CAC template strand from a dG•A(template) misinsertion that is subsequently stabilized by a flanking dG•C(template) pairing. Alternatively, the same mutation could arise on the GTG template strand from a dC•T(template) misinsertion stabilized by a flanking dC•A(template) pairing. The ratios of incorrect to correct dNTP concentrations would play a critical role in this process. While caution is warranted given the relatively small number of mutations, it does raise the possibility that altered dNTP pools could be playing a role in creating this mutation spectrum. In The Cancer Genome Atlas database of human tumors (*n* = 11 125) there is a single patient with mutation at the conserved Y285 residue (TCGA-DDEI, *RRM1*-Y285C). The mutation spectrum from this single patient is also defined by two peaks, one of which is also T > G-GTG. The total number of mutations in this tumor sample is only 113, which supports the notion that *RRM1*-Y285C mutation does not lead to a strong mutator phenotype. The COSMIC SBS89 mutation signature has an unknown aetiology and is defined primarily by three elements: C > T-ACW, T > A-CTN and a single sharp peak at T > G-GTG. This T > G-GTG is unusual and resembles the significant peak seen in the *Rrm1*^+/Y285A^ mice. An interesting possibility worth further study is that tumors with SBS89 signature may in some part have an Rrm1-mediated nucleotide imbalance. Our assessment of hRRM1-Y285C protein activity in RNR assays revealed even greater impairment in dGDP and dADP production compared with hRRM1-Y285A (Supplementary Figure S2), indicating an even stronger reduction in dGTP and dATP pools and potential contribution to replication stress and genome instability in this tumor context.

Several other residues located in the allosteric specificity site of *hRRM1*, including A283, R284, D287, R293 and P294, have also been identified as mutated in human cancer samples. Among these, mutations in A283, D287 and R283 have been shown to affect budding yeast RNR activity (9,10), and will most likely negatively impact dNTP pools in human cells. Moreover, cancer cells are reported to have multiple mutations in other regions of *hRRM1*, as well as in other RNR subunits, *RRM2* and *RRM2B*, that possibly also can negatively impact dNTP pools. Earlier studies demonstrated that broad overexpression of *Rrm2* or *Rrm2b* induces lung neoplasms in transgenic mice (56). However, it was not established whether this overexpression led to altered dNTP pools, and an alternative carcinogenic mechanism involving the formation of reactive oxygen species was suggested. While the prevalence of mutations leading to decreased dNTP availability remains unknown, the presence of numerous proteins directly involved in *de novo* dNTP synthesis, degradation and salvage in mammalian cells underscores the potential for alterations in dNTP pools. Moreover, proteins involved in *de novo* synthesis or salvage of ribonucleotides, which serve as substrates for *de novo* dNTP synthesis, further add complexity to this landscape. For instance, in budding yeast, inactivation of URA7, which encodes CTP synthase, leads to a significant reduction in CTP levels, subsequently negatively impacting dCTP synthesis (57). Investigating the prevalence of mutations affecting dNTP pools in these genes could provide valuable insights into tumorigenesis.

In summary, this study sheds light on the intricate interplay between nucleotide metabolism, replication stress and tumorigenesis in the mouse model. By delineating the role of dNTP pool imbalances in fuelling replication stress-induced tumorigenesis, our findings provide novel insights into the mechanisms underlying cancer development. Additionally, they offer potential avenues for therapeutic intervention aimed at inhibition of dNTP synthesis further increasing replication stress and genomic instability in cancer cells with defective dNTPs production.

## Supplementary Material

gkae843_Supplemental_File

## Data Availability

Whole exome sequencing data SRA accession number: PRJNA1114130.

## Notes

Present address: Leonard G. Williams, Informuta, New Orleans, LA, USA.
