# Supplementary material for: Altered dNTP pools accelerate tumor formation in mice
Source: Nucleic Acids Res. 2024 Oct 3;52(20):12475–86. doi: 10.1093/nar/gkae843 (PMC11551754; doi:10.1093/nar/gkae843)
Supplement: gkae843_Supplemental_File [file gkae843_supplemental_file.pdf]

SUPPLEMENTARY FIGURES AND LEGENDS

A

S. cerevisiae	283	-ARYVDQGGNKRPGAFA-298
M. musculus	283	-ARYVDQGGNKRPGAFA-298
H. sapiens	283	-ARYVDQGGNKRPGAFA-298

283 284 285 287 293 294

B

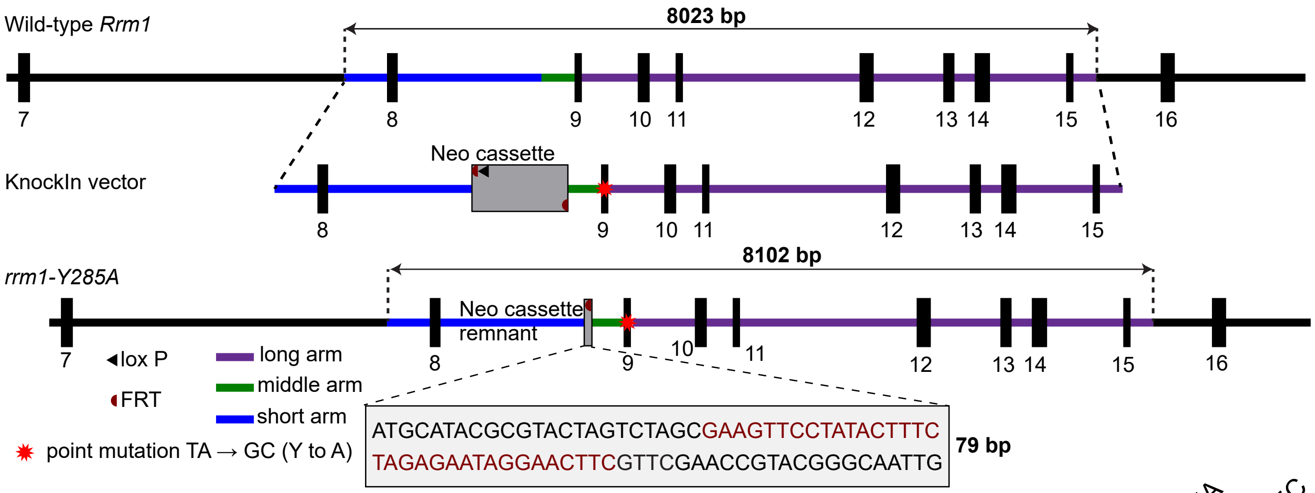

C

Rrm1$^{+/Y285A}$ × *Rrm1*$^{+/Y285A}$

Genotypes	Expected distribution	Observed distribution
Rrm1$^{+/+}$	1	1.0 (n = 39)
Rrm1$^{+/Y285A}$	2	1.7 (n = 68)
Rrm1$^{Y285A/Y285A}$	1	0.0 (n = 0)

D

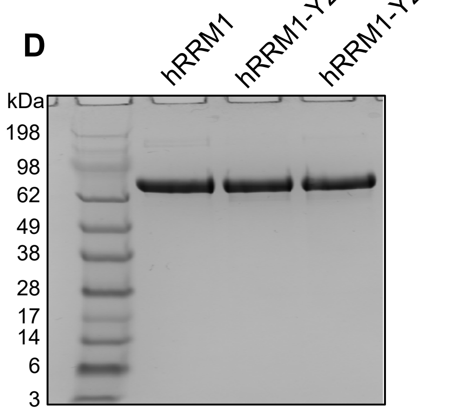

Figure S1. (A) Alignment of protein sequences showing the allosteric specificity sites of RNRs from *S. cerevisiae, M. musculus, and H. sapiens.* Numbered residues indicate those found mutated in hRRM1 in cancer samples. (B) Illustration of the strategy employed for constructing the *rrm1*-Y285A knock-in allele. (C) Presentation of outcomes resulting from the cross between *Rrm1*$^{+/Y285A}$ × *Rrm1*$^{+/Y285A}$ mice. (D) SDS-PAGE analysis of purified recombinant hRRM1, hRRM1-Y285A, and hRRM1-Y285C proteins.

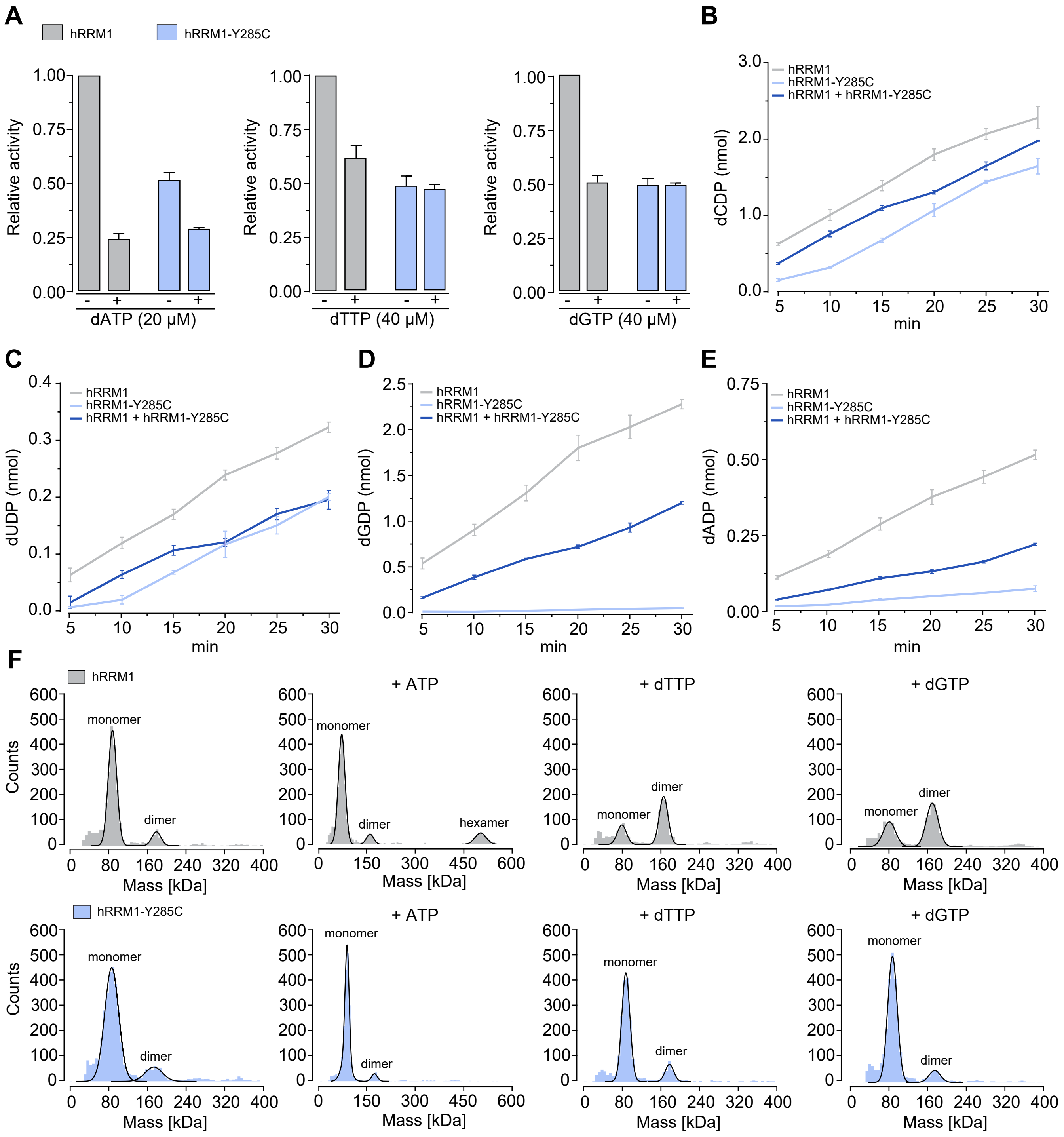

Figure S2. Activity and subunit composition of hRRM1 and hRRM1-Y285C in the presence of different allosteric effectors. (A) Relative CDP-reducing activity of hRRM1 and hRRM1-Y285C proteins with 2 mM ATP, with or without 20 µM dATP, 40 µM dTTP or 40 µM dGTP. Activity of hRRM1 in the presence of ATP is normalized to 1. (B-E) Activity of hRRM1, hRRM1-Y285C, and a 1:1 mixture of hRRM1/hRRM1-Y285C protein mixture with four substrates (200 µM each of CDP, UDP, GDP and ADP) and four allosteric effectors at physiological concentrations (2 mM ATP, 30 µM dTTP, 14 µM dATP and 6 µM dGTP) over a 30-minute time course. (F) Mass-photometry analysis of the subunit composition of hRRM1 and hRRM1-Y285C proteins in the presence or absence of positive allosteric effectors (2 mM ATP, 100 µM dTTP and 100 µM dGTP).

Supplementary Table 1.

Oligonucleotides	Sequences (5'to 3')
hRRM1-Y285C-F	GTAGGTCTCGCACGT**TGT**GTTGATCAAGGTGGTAATAAAC
hRRM1-Y285A-F	GTAGGTCTCGCACGT**GCA**GTTGATCAAGGTGGTAATAAAC
hRRM1-285-R	GTAGGTCTCACGTGCGGTATTGTTATAAACACGC
NDEL1	GCCACAGCCCACTTATCTTTTCAAA
GT1	GTAAAACAATATATAACTGAACCAAGTAG
